# Development of Sequential Fermentation Starters and Comparison of Quality Characteristics for Black Barley Vinegar Production

**DOI:** 10.3390/microorganisms13112576

**Published:** 2025-11-12

**Authors:** Soo-Young Lee, Hyun-wook Jang, Hee-Min Gwon

**Affiliations:** Fermented and Processed Food Research Division, Department of Food Sciences, NICS, RDA, Wanju 55365, Republic of Korea; sylee5694@korea.kr (S.-Y.L.); jhj4676@korea.kr (H.-w.J.)

**Keywords:** vinegar, black barley, acetic acid fermentation, sequential fermentation starters

## Abstract

To identify the optimal sequential starter and fermentation conditions, black barley vinegar (BBV) was produced by stepwise inoculation with two acetic acid bacteria (AAB). The optimal fermentation conditions for BBV12-8 (fermentation with *Acetobacter ascendens* GV-12 followed by *A. ascendens* GV-8) were pH 3.0 and 20 °C, and for BBV8-22 (fermentation with *A. ascendens* GV-8 followed by *Acetobacter pasteurianus* GV-22) were pH 3.0 and 30 °C. During fermentation, the levels of most organic acids decreased in both vinegars, while acetic acid increased significantly. Total free amino acids also decreased, and taste fingerprint analysis revealed increased sourness and umami and decreased saltiness with fermentation. In particular, BBV12-8 exhibits higher ethyl acetate and 3-methylbutyl acetate than BBV8-22. This difference was closely related to fermentation temperature, as highly volatile esters were better preserved at 20 °C than at 30 °C. Therefore, *A. ascendens* GV-12 followed by *A. ascendens* GV-8 sequential inoculation at pH 3.0 and 20 °C was selected as the optimal condition for producing black barley vinegar with improved flavor characteristics, suggesting that sequential AAB inoculation can be applied to produce vinegar with enhanced flavor quality.

## 1. Introduction

Vinegar is produced by oxidizing ethanol to acetic acid through the activity of acetic acid bacteria (AAB) after the alcoholic fermentation of sugar- or starch-containing substrates by yeasts [1]. During this acetic fermentation process, various bioactive compounds are generated, and vinegar has been reported to possess functional properties such as antioxidant and anti-obesity activities [2,3]. In particular, acetic acid contained in vinegar inhibits the growth of spoilage microorganisms and acts as an effective preservative that contributes to preventing food spoilage [4]. In this context, the development of functional vinegars using diverse raw materials, in addition to cereals and fruits, has recently expanded [5,6,7,8].

The production of vinegar and other fermented foods and beverages involves complex microbial communities; therefore, understanding the associated microbiota is crucial [9]. The main AAB involved in vinegar production include *Acetobacter*, *Komagataeibacter*, and *Gluconobacter* [10]. Depending on the bacterial strain, differences in acid, sugar, and alcohol tolerance, as well as in acid-producing capacity, can influence the quality of the final product [11,12]. Moreover, considerable variations in biological activities—such as antimicrobial, antioxidant, and angiotensin-converting enzyme (ACE) inhibitory activities—have been reported among strains, which are directly related to the functional properties of vinegar [13,14]. Traditional vinegar fermentation, which involves diverse microorganisms, requires a long acetification period. In contrast, the use of defined starter cultures can shorten this period and improve process standardization and product reproducibility [15].

Recently, increasing attention has been paid to acetic fermentations using multiple AAB strains. Previous studies examined the acid-producing ability and volatile aroma profiles of different AAB combinations [16]. It was reported that mixed inoculation reached the target acidity faster and produced a wider spectrum and higher contents of volatile aroma compounds compared with single-strain inoculation. However, most of these evaluations were performed in culture media rather than in a traditional two-stage process consisting of alcoholic and acetic fermentations.

Therefore, in this study, black barley makgeolli was prepared through alcoholic fermentation and then subjected to acetic fermentation by sequentially inoculating two AAB strains to produce black barley vinegar. Makgeolli, a traditional Korean rice wine with an alcohol content of approximately 6–8%, provides an appropriate substrate for acetic fermentation [17]. Black barley is rich in polyphenols and anthocyanins and thus has high potential as a functional raw material; however, studies on fermented beverages utilizing black barley remain limited [18,19,20]. Furthermore, the optimization of starter culture conditions for vinegar production has been reported to improve the quality of vinegar [21].

Accordingly, acetic fermentation was conducted under various pH and temperature combinations, and changes in physicochemical properties, organic acids, free amino acids, taste fingerprints, and volatile compounds during fermentation were compared and analyzed. Particular emphasis was placed on aroma characteristics that determine vinegar quality, with the aim of identifying the optimal sequential starter combination and fermentation conditions. This study provides insights into the potential application of sequential AAB inoculation for vinegar production.

## 2. Materials and Methods

### 2.1. Black Barley Makgeolli Preparation

For the preparation of black barley makgeolli, black barley and corn flour were used as the main and sub-ingredients, respectively. The black barley used was the ‘heukdahyang’ variety, a hull-less black barley developed by the National Institute of Crop Science (Wanju, Republic of Korea). Based on previous study, nuruk was produced by co-inoculating sterilized wheat and mung beans with *Aspergillus oryzae* SU-Y, *Lichtheimia ramosa* KJ-WF, and *Aspergillus niger* JA-B, and a starter mash (also referred to as seed mash) produced with *Saccharomyces cerevisiae* Y204 was used [22]. Nuruk is a fermentation agent used in the production of makgeolli, a traditional turbid rice wine in Korea. Fungi in nuruk saccharify rice starch to glucose, and the yeast in starter mash subsequently ferments this glucose to ethanol and carbon dioxide [23].

Black barley (20 kg) was washed, soaked in water for 16 h, drained, and then steamed for 1 h to prepare steamed black barley rice ‘godubap’. After cooling, the steamed black barley, corn flour (2 kg), and nuruk (2 kg) were placed in a stainless-steel fermentation vessel, followed by the addition of 36 L of water and thorough mixing. To prevent contamination by undesirable microorganisms during the initial fermentation stage, 100 mL of lactic acid (FUJIFILM Wako Pure Chemical Co., Osaka, Japan) was added. A starter mash (200 mL) was added, and the mixture was allowed to ferment at 25 °C. After 7 days, makgeolli with 10% (*v*/*v*) alcohol content was filtered through a mesh sieve and centrifuged. The supernatant was then adjusted to 6% (*v*/*v*) alcohol content and used for seed vinegar preparation and black barley vinegar production.

### 2.2. Seed Vinegar Preparation

Three acetic acid bacteria strains—*Acetobacter ascendens* GV-8, *A. ascendens* GV-12, and *Acetobacter pasteurianus* GV-22—were selected from isolates obtained by the Fermented Food Division of the National Institute of Crop Science, Rural Development Administration, Republic of Korea. Under fermentation conditions at 30 °C, these strains exhibited high acetic acid productivity, achieving titratable acidity values of 5.33 ± 0.19 (GV-8), 5.62 ± 0.03 (GV-12), and 5.40 ± 0.01 (GV-22) (as acetic acid equivalents) [16]. In addition to high acetic-acid productivity, *A. ascendens* GV-12 demonstrated alcohol tolerance at 8% (*v*/*v*) ethanol, *A. ascendens* GV-8 and *A. pasteurianus* GV-22 have been reported to possess acid tolerance at pH 4.0. The acetic acid bacteria stocks were activated by streaking on GYCE agar medium (3% (*w*/*v*) glucose, 0.5% (*w*/*v*) yeast extract, 1% (*w*/*v*) CaCO_3_, 5% (*v*/*v*) ethanol, and 2% (*w*/*v*) agar) and incubating at 30 °C for 3 days. The activated strains were then inoculated into a selective medium prepared in this study (0.5% (*w*/*v*) glucose, 0.5% (*w*/*v*) yeast extract, 1% (*w*/*v*) glycerin, 0.02% (*w*/*v*) magnesium sulfate heptahydrate (MgSO_4_·7H_2_O), 4% (*v*/*v*) ethanol, and 1% (*v*/*v*) acetic acid), followed by subculturing and scale-up every 3 days to prepare 200 mL of acetic acid bacteria culture. To prevent off-flavors caused by the culture broth, the acetic acid bacteria culture was centrifuged at 8000 rpm for 15 min (CR22GIII, Hitachi Co., Tokyo, Japan). The resulting pellet was washed with 1X PBS buffer (Gibco, Thermo Fisher Scientific, Waltham, MA, USA) and centrifuged again to remove the buffer. The pellet was then resuspended in adjusted supernatant of black barley makgeolli to prepare the seed vinegar.

The prepared seed vinegar was transferred into a jar covered with sterilized cotton cloth and a lid, and acetic acid fermentation was carried out at 30 °C. The mixture was intermittently stirred until a pellicle formed on the surface. After pellicle formation, acidity was measured to confirm proper fermentation, and fermentation continued until the acidity reached above 5%.

### 2.3. Production of Black Barley Vinegar Using Sequential Starters and Selection of Fermentation Conditions

To determine the optimal pH and temperature for acetic acid fermentation, black barley makgeolli was adjusted to pH 3.0 or pH 4.0. It was then inoculated with a seed vinegar culture at 10% (*v*/*v*) relative to the makgeolli volume. All fermentations used a 2.0 L working volume in 4.0 L fermentation vessels. Each vessel was covered with a sterilized cotton cloth to allow adequate air exchange. Fermentations were then conducted at 20 °C or 30 °C, yielding four combinations (pH 3.0/20 °C, pH 3.0/30 °C, pH 4.0/20 °C, pH 4.0/30 °C). After 4 days, a second seed vinegar culture was added at 10% (*v*/*v*) relative to the working-broth volume to continue the fermentation. BBV12–8 denotes sequential inoculation with *A. ascendens* GV-12 followed by *A. ascendens* GV-8, whereas BBV8–22 denotes *A. ascendens* GV-8 followed by *A. pasteurianus* GV-22. For each condition, fermentations were performed in three independent biological replicates, and all measurements were conducted in technical triplicate.

During fermentation, samples were collected every 5 days to measure alcohol content and total acidity, capturing early–mid–late acetification phases while minimizing vessel opening and contamination. Fermentation lasted 20 days in total, and the collected samples were stored at −80 °C. The completion of acetic acid fermentation was defined as the point when alcohol was fully consumed and total acidity reached over 5%. For BBV12-8, fermentation was completed on day 10 at pH 3 and 20 °C, and for BBV8-22, fermentation was completed on day 10 at pH 3 and 30 °C, based on these criteria. Subsequently, analyses of total polyphenols, organic acids, free amino acids, and volatile compounds were conducted using samples collected at the initial fermentation stage (day 0) and at fermentation completion (day 10) under each respective condition.

### 2.4. Physicochemical Properties

The physicochemical properties of black barley vinegar were evaluated by analyzing alcohol content and total acidity. Alcohol content was measured using a distillation method adapted from a standard protocol. Vinegar samples (100 mL) were diluted with 40 mL of distilled water and then distilled using a VAPODEST 200 apparatus (C. Gerhardt GmbH & Co., Königswinter, Germany). After collecting 80 mL of the distillate, 20 mL of distilled water was added to bring the volume to 100 mL. The alcohol concentration was then measured at 15 °C using a DMA 5000 M densitometer (Anton Paar Co., Graz, Austria), with the instrument’s automatic temperature control. Results were reported as % (*v*/*v*) [24].

For total acidity analysis, 1 mL of vinegar sample was diluted tenfold with 9 mL of distilled water and used as the test solution. Titration was performed using 0.1% phenolphthalein (Daejung Co., Siheung, Republic of Korea) as an indicator and 0.1 N NaOH solution (Daejung Co.) as the titrant. pH was measured at room temperature (20 °C) using an Orion 3 Star pH meter (Thermo Fisher Scientific, Beverly, MA, USA). Samples were titrated with 0.1 N NaOH until a final pH of 8.30 was reached. The volume of NaOH consumed was converted to acetic acid concentration (%) to calculate total acidity [25].

### 2.5. Organic Acids

Organic acid analysis was conducted by the Kyungpook national university center for research facilities (Daegu, Republic of Korea). Vinegar samples were centrifuged at 10,000 rpm for 10 min using a ScanSpeed mini centrifuge (LaboGene, Lillerød, Denmark), and the supernatant was filtered through a 0.2 μm membrane filter (25CS020AS, Advantec Toyo Kaisha, Ltd., Tokyo, Japan) for use as the analysis sample. High-performance liquid chromatography (HPLC) analysis was performed using a Prominence HPLC system (Shimadzu Co., Kyoto, Japan) equipped with a PL Hi-Plex H column (8 μm, 7.7 × 300 mm, Agilent Technologies, Santa Clara, CA, USA) maintained at 65 °C. The mobile phase consisted of 0.005 M H_2_SO_4_ in water, with a flow rate of 0.6 mL/min. A 20 μL sample volume was injected, and organic acids were detected using an RID-10A refractive index detector (Shimadzu Co.). Standard solutions of citric acid, malic acid, succinic acid, lactic acid, acetic acid, propionic acid, and butyric acid were prepared by dissolving each in 0.1 N HCl solution. Quantification was performed using standard curves constructed for each organic acid. Peaks in the sample chromatograms were identified and confirmed by comparing retention times with those of the standard organic acids.

### 2.6. Free Amino Acids

Free amino acid analysis was conducted by the Kyungpook national university center for research facilities. 1 mL of vinegar samples were mixed with 1 mL of 0.5% trichloroacetic acid and left to stand for 1 h to precipitate proteins. The mixture was then centrifuged at 10,000 rpm for 10 min, and the supernatant was filtered through a 0.2 μm membrane filter. Quantification was performed using an Amino Acid Autoanalyzer (LA8080, Hitachi Co., Tokyo, Japan). An ion exchange column was used for separation, with the reaction column maintained at 135 °C. Ninhydrin reagent (FUJIFILM Wako Pure Chemical Co.) was employed as the chromogenic agent, and detection was carried out at dual wavelengths of 570 nm and 440 nm. The mobile phase flow rate was set to 0.3 mL/min, and the analysis time per sample was approximately 157.3 min.

### 2.7. Taste Fingerprint

The taste fingerprint of the black barely vinegars was analyzed using an electronic tongue (Astree II, Alpha MOS, Toulouse, France) [26]. To adjust acidity and prevent sensor saturation, each sample was diluted 1:100 with distilled water, and the diluted solutions were continuously stirred at room temperature during measurement [27]. To prevent cross-contamination between samples, the autosampler was rinsed with purified water before and after each measurement. Each sample was measured seven times, and the mean value of the middle three measurements (excluding the first and last two) was used for analysis. The results were expressed as taste attributes, including saltiness (CTS), sourness (AHS), and umami (NMS), and were converted into relative taste scores ranging from 0 to 10 according to the response range of each sensor. These scores were then used to evaluate the overall taste patterns. Data analysis was performed using AlphaSoft 17 software (Alpha MOS, Toulouse, France).

### 2.8. Volatile Compounds

Volatile compound analysis was conducted by EZMASS (Jinju, Republic of Korea). Vinegar samples were diluted fivefold with triple-distilled water, and 2 mL of triple-distilled water containing the internal standard 2-methyl-1-pentanol was added to 1 mL of the diluted vinegar sample. The resulting 3 mL mixture was placed in a headspace vial and stirred at 550 rpm for 20 min at room temperature. Volatile compounds were adsorbed for 5 min using solid-phase microextraction (SPME) and then injected into a GC/MS system. Analysis was performed using a GC-2010 Plus and GCMS-TQ 8030 (Shimadzu Co.) equipped with a DB-WAX column (30 m × 0.25 mm, 0.25 μm, J&W Scientific, Folsom, CA, USA). The injection volume was 1 μL, the injection temperature was set at 230 °C, and helium was used as the carrier gas at a flow rate of 1 mL/min. The oven temperature program started at 40 °C for 2 min, increased to 100 °C at 5 °C/min, then ramped to 240 °C at 20 °C/min, maintaining 240 °C for 4 min. The mass spectrometer (MS) operated in Q3 scan mode, with an ion source temperature of 200 °C, interface temperature of 250 °C, detector voltage of 0.1 kV, event time of 0.03 s, and electron energy of 15 eV. Volatile compounds were identified solely using the NIST 11 and Wiley 9 mass spectral libraries.

### 2.9. Statistical Analysis

All experiments were performed in triplicate, and results are presented as mean ± SD. Statistical analysis was conducted using SPSS software (ver. 29.0, SPSS Inc., Chicago, IL, USA). Comparisons among three or more groups were performed by one-way analysis of variance (ANOVA), and significant differences between means were determined by Duncan’s new multiple range test at *p* < 0.05. For comparisons between two groups, an independent samples *t*-test (Student’s *t*-test) was used. Prior to parametric testing (ANOVA/*t*-test/Duncan), data were assessed for normality (Shapiro–Wilk) and homogeneity of variance (Levene). When normality was not met, variables were transformed (e.g., log10) prior to analysis. Discriminant Function Analysis (DFA) was performed to visualize and analyze patterns in taste fingerprint data. For volatile compound data, Principal Component Analysis (PCA), Partial Least Squares Discriminant Analysis (PLS-DA), Variable Importance in Projection (VIP), and permutation tests were performed using SIMCA-P+ version 18 (Umetrics, Umeå, Sweden). For PCA/PLS-DA, variables were autoscaled (mean-centered, unit variance).

## 3. Results and Discussion

### 3.1. Production of Black Barley Vinegar Using Sequential Starters and Selection of Optimal Fermentation Conditions

To investigate the changes in quality characteristics of black barley vinegar produced using sequential starters under different acetic acid fermentation conditions and to determine the optimal fermentation conditions, acetic acid fermentation was conducted under four conditions (pH 3 or pH 4, 20 °C or 30 °C). Acetic acid bacteria oxidize ethanol to acetic acid under aerobic conditions; therefore, the progression of fermentation and bacterial growth can be monitored by the decrease in alcohol content and the increase in total acidity during acetic acid fermentation [28,29]. In this study, the end of acetic acid fermentation was defined as the time point when total acidity reached 5% or higher and alcohol was completely depleted.

**Figure 1:**
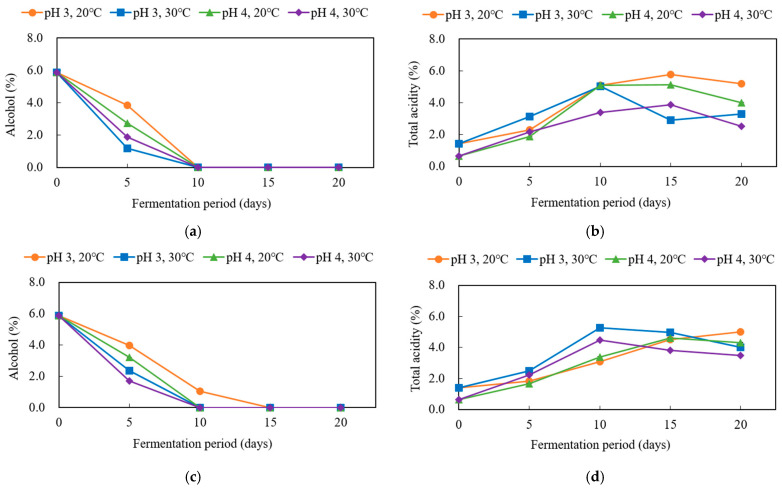
Changes in alcohol content (**a**,**c**) and total acidity (**b**,**d**) of black barley vinegar during sequential fermentation by stepwise inoculation of multiple AAB under different pH, temperatures, and periods. Samples: (**a**,**b**) BBV12-8; (**c**,**d**) BBV8-22.

As a result, BBV12-8, produced by inoculation of *A. ascendens* GV-12 followed by *A. ascendens* GV-8 after 4 days, reached a total acidity of 5% and complete alcohol oxidation on day 10 of fermentation at pH 3 and 20 °C. Similarly, BBV8-22, produced by inoculation of *A. ascendens* GV-8 followed by *A. pasteurianus* GV-22 after 4 days, achieved these fermentation endpoints at pH 3 and 30 °C on day 10 (Figure 1). In addition, these fermentation conditions demonstrated stable maintenance of total acidity over the fermentation period compared to other conditions (Figure 1b,d). Therefore, these conditions were selected as the optimal fermentation conditions for each sequential starter.

### 3.2. Organic Acids

During acetic acid fermentation, the contents of citric acid, malic acid, succinic acid, and lactic acid generally decreased. In particular, lactic acid markedly declined from 12.04 ± 0.51 mg/100 mL to 1.84 ± 0.01 mg/100 mL in BBV12-8 and from 12.12 ± 0.05 mg/100 mL to 1.52 ± 0.25 mg/100 mL in BBV8-22. Conversely, acetic acid increased substantially from 39.79 ± 3.09 mg/100 mL to 408.09 ± 1.45 mg/100 mL in BBV12-8 and from 39.26 ± 0.05 mg/100 mL to 423.53 ± 25.74 mg/100 mL in BBV8-22 (Table 1). Consequently, the total organic acid content on day 10 reached 530.56 ± 1.81 mg/100 mL in BBV12-8 and 550.36 ± 27.02 mg/100 mL in BBV8-22.

Vinegar is known to exhibit various beneficial effects, including antimicrobial and antioxidant activities, as well as antidiabetic, antitumor, anti-obesity, and cardiovascular protective effects [30]. In particular, acetic acid, the major organic acid in vinegar, has been reported to exert strong antimicrobial activity against pathogenic microorganisms such as *Escherichia coli* O157:H7. For instance, treating shredded lettuce inoculated with *E. coli* O157:H7 with 5% acetic acid (pH 3.0) for 5 min reduced the bacterial population by approximately 3 log units [31]. Among acetic, lactic, malic, and citric acids, acetic acid has been shown to be the most effective at inhibiting the growth and colony formation of *E. coli* O157:H7 [32]. Furthermore, the anti-obesity activity of aronia vinegar increased sharply during fermentation, primarily attributable to the rapid rise in acetic acid content [33]. Therefore, black barley vinegar produced in this study is also expected to exhibit similar functional properties, including antimicrobial and anti-obesity effects, due to the substantial accumulation of acetic acid during fermentation.

### 3.3. Free Amino Acids

**Figure 2:**
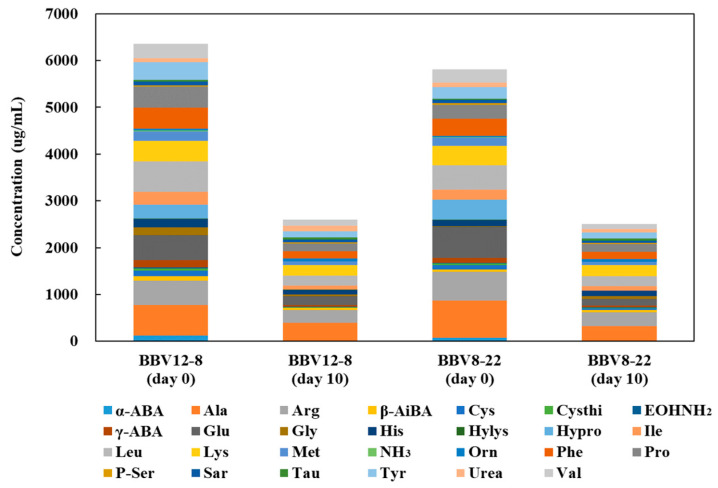
Changes in free amino acid content of black barley vinegar during sequential fermentation by stepwise inoculation of multiple AAB. Symbols: α-ABA, L-α-Amino-n-butyric acid; Ala, L-Alanine; Arg, L-Arginine; β-AiBA, β-Aminoisobutyric acid; Cys, L-Cystine; Cysthi, L-Cystathionine; EOHNH_2_, Ethanolamine; γ-ABA, γ-Aminobutyric acid; Glu, L-Glutamic acid; Gly, L-Glycine; His, L-Histidine; Hylys, D,L- and allo-Hydroxylysine; Hypro, Hydroxyproline; Ile, L-Isoleucine; Leu, L-Leucine; Lys, L-Lysine; Met, L-Methionine; NH_3_, Ammonia; Orn, L-Ornithine; Phe, L-Phenylalanine; Pro, L-Proline; P-Ser, D,L-O-Phosphoserine; Sar, Sarcosine; Tau, Taurine; Tyr, L-Tyrosine; Urea, Urea; Val, L-Valine.

In BBV12-8, the total free amino acid content decreased from 6354.30 ± 185.24 μg/mL at day 0 of acetic acid fermentation to 2430.11 ± 360.86 μg/mL at day 10, and in BBV8-22, the contents were 5736.28 ± 499.31 μg/mL and 2509.11 ± 175.54 μg/mL, respectively (Figure 2; Table S1). This decrease is likely due to the utilization of free amino acids as energy and nitrogen sources by microorganisms during fermentation, as well as their conversion into volatile compounds or secondary metabolites through protein hydrolysis and amino acid transformation pathways [34,35].

Residual yeasts and associated microbiota can catabolize amino acids via the Ehrlich pathway, in which amino acids are first transaminated to the corresponding α-keto acids, followed by decarboxylation and reduction to form higher alcohols and their derived esters [36]. This metabolic conversion not only decreases the pool of free amino acids but also contributes to the generation of key volatile aroma compounds such as fusel alcohols and esters, thereby shaping the aroma profile of the vinegar.

In parallel, changes in the concentrations of taste-active, nonvolatile free amino acids modulate the basic taste attributes. Amino acids and their derivatives generated via protein hydrolysis and transformation during fermentation contribute to the formation of umami, sweetness, and bitterness, playing an important role in the taste and overall quality of the final vinegar product [37]. In particular, both BBV12-8 and BBV8-22 showed decreases in glutamic acid (umami), alanine and proline (sweetness), and arginine and leucine (bitterness). These changes are presumed to contribute to a cleaner and smoother flavor profile of the vinegar during fermentation [38].

### 3.4. Taste Fingerprint

**Figure 3:**
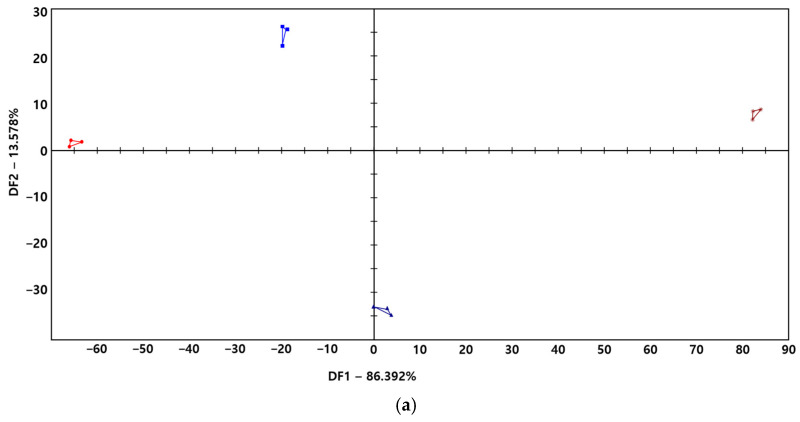

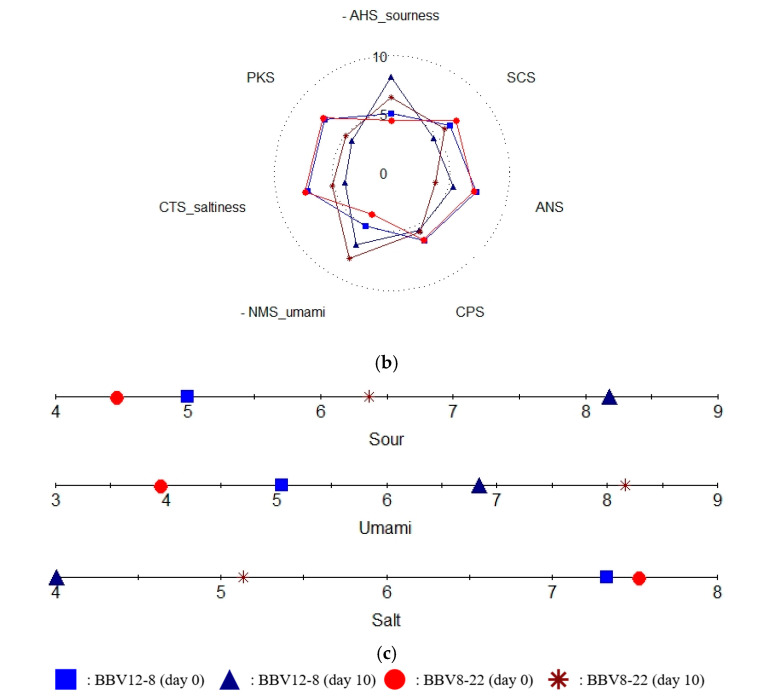
Variation in taste fingerprints in black barley vinegars analyzed in this study: (**a**) Discriminant Function Analysis (DFA) score plot; (**b**) overall variation in sensory characteristics; (**c**) intensity of individual sensory characteristics.

The results of the taste fingerprint analysis of black barley vinegar are presented in Figure 3. The DFA plot showed that DF1 and DF2 accounted for 86.392% and 13.578% of the discriminating power of the model, respectively (Figure 3a). In the DFA score plot, BBV12-8 and BBV8-22 at day 0 clustered on the negative side of DF1, whereas both vinegars at day 10 shifted to the positive side of DF1.

Furthermore, discriminative fingerprints were observed in the sensory characteristics (Figure 3b,c). Sourness (AHS) ranged from 4.5 to 8.2, with BBV12-8 at day 0 showed an intensity of 5.0. Whereas BBV12-8 at day 10 exhibiting a high intensity of 8.2. BBV8-22 at day 0 showed an intensity of 4.5. Whereas BBV8-22 at day 10 exhibited an intensity of 6.4. This finding is consistent with previous studies reporting that the sourness intensity of vinegar increases with aging period [26]. Compared with sourness, a similar tendency was observed for umami (NMS). BBV12-8 and BBV8-22 at day 0 showed intensities of 5.0 and 3.9, whereas both vinegars at day 10 showed an intensity of 8.1 and 6.8, respectively. However, the opposite tendency was observed for saltiness (CTS). BBV12-8 and BBV8-22 at day 0 showed intensities of 7.5 and 7.4, respectively, whereas both vinegars at day 10 showed an intensity of 4.0 and 5.1, respectively. These findings are valuable for understanding overall taste changes and taste balance in vinegar and may be further applied to quality control and prediction of sensory characteristics in vinegar products.

### 3.5. Volatile Compounds

**Figure 4:**
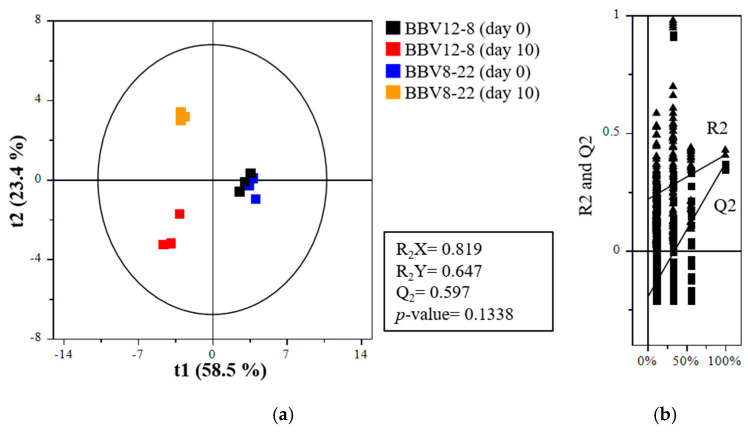
Partial least squares discriminant analysis (PLS-DA) of black barley vinegar during sequential fermentation by stepwise inoculation of multiple AAB: (**a**) PLS-DA score plot; (**b**) permutation test.

**Figure 5:**
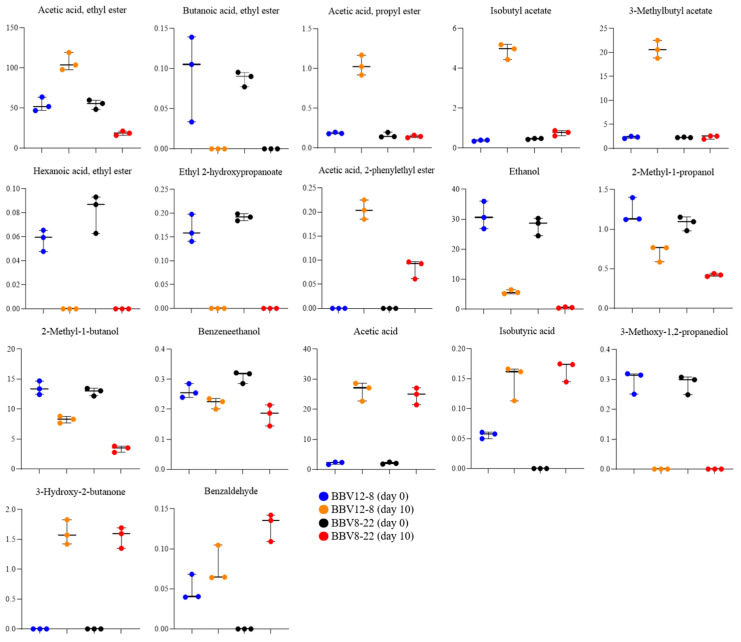
Boxplots of major volatile compounds in black barley vinegar during sequential fermentation by stepwise inoculation of multiple AAB.

**Figure 6:**
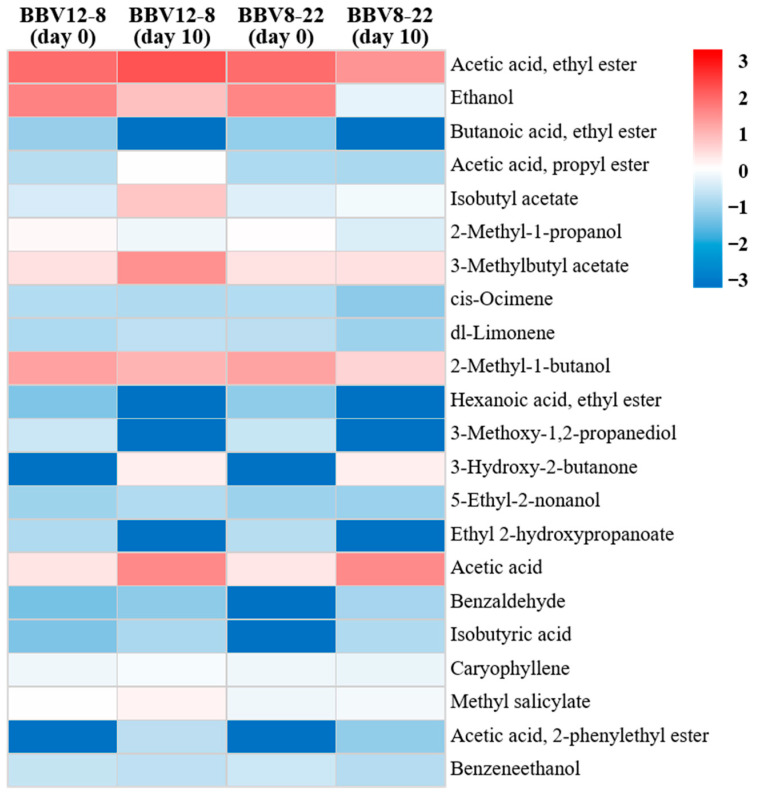
Heatmap of major volatile compounds in black barley vinegar during sequential fermentation by stepwise inoculation of multiple AAB.

Aroma is one of the most critical quality indicators of vinegar, serving as a key factor that determines consumer preference and acceptability [39]. The composition of aroma compounds varies depending on raw materials, microbial communities, microbial metabolism, and fermentation conditions [40,41,42,43,44,45]. Therefore, precise analysis of the composition and dynamics of aroma-active compounds is essential for a systematic evaluation of vinegar flavor quality. The volatile compound profiles of the black barley vinegar were analyzed by GC-MS, and the results are presented in Table 2 and Table 3, and Figure 4, Figure 5 and Figure 6. Table 2 summarizes the major metabolites with significant statistical relevance and high variable importance in projection (VIP) scores. Table 3 shows the relative concentrations (area values) of each major volatile compound. Figure 4 shows the results of partial least squares discriminant analysis (PLS-DA) and a permutation test. Figure 5 presents boxplots illustrating the concentration distributions and inter-sample differences in individual aroma compounds. Figure 6 displays a heatmap generated to visualize volatile com-pounds that exhibited statistically significant differences (*p* < 0.05).

Among the major aroma compounds in BBV12-8 and BBV8-22, ethyl acetate (acetic acid ethyl ester) was present at similar levels before fermentation in both vinegars; however, after acetic acid fermentation, it increased by more than two-fold in BBV12-8 but decreased by more than half in BBV8-22. Consequently, on day 10 of fermentation, the total aroma compound concentration in BBV12-8 (179.42) was more than three times higher than that of BBV8-22 (54.57) (Table 3). Notably, ethyl acetate, a representative ester contributing fruity notes, was either more actively produced or better retained in BBV12-8, representing a key marker for the aroma difference between the two vinegars [46,47]. Additionally, 3-methylbutyl acetate, which imparts a banana-like aroma, showed significantly higher levels in BBV12-8 (*p* < 0.05), contributing to the improved flavor characteristics of this vinegar [48]. Both ethyl acetate and 3-methylbutyl acetate are known as major aroma compounds in most fermented vinegars, greatly influencing vinegar flavor quality [49,50,51]. These results align with a previous study reporting that levels of volatile flavor compounds were higher when acetic acid fermentation was conducted at 20 °C than at 30 °C using sequential starters [16]. This pattern appears to be closely related to fermentation temperature. Among volatile compounds generated via the Ehrlich pathway, highly volatile esters were likely better preserved at 20 °C than at 30 °C [36,52].

Ethanol concentrations decreased after acetic acid fermentation in both BBV12-8 and BBV8-22, while acetic acid, the main organic acid in vinegar, increased substantially, confirming normal acetic acid fermentation by acetic acid bacteria (Figure 5 and Figure 6). In particular, ethanol levels dropped sharply to nearly undetectable amounts in BBV8-22 after fermentation, suggesting either a higher efficiency of ethanol conversion to acetic acid or greater ethanol volatilization loss during fermentation in BBV8-22. Because the optimal growth temperature of acetic acid bacteria is around 25–30 °C, BBV8-22 fermented at 30 °C probably underwent more vigorous ethanol oxidation during the acetification process, which in turn led to greater hydrolysis of ethyl acetate [53].

Taken together, the sequential starter composed of *A. ascendens* GV-12 followed by *A. ascendens* GV-8 was determined to be the most suitable combination for enhancing the flavor characteristics of black barley vinegar. Accordingly, this sequential starter and the fermentation condition of pH 3.0 at 20 °C were selected as the optimal parameters.

## 4. Conclusions

In this study, black barley vinegar was produced by the sequential inoculation of multiple acetic acid bacteria (AAB) into black barley makgeolli, and acetic acid fermentation was conducted under various conditions to compare and analyze the quality characteristics, aiming to select the optimal sequential starter combination and fermentation conditions for black barley vinegar production. Sequential starters not only facilitated efficient acetic acid fermentation but also improved flavor characteristics by modulating organic acids, free amino acids, and aroma profiles. Notably, at pH 3.0 and 20 °C, the combination of *A. ascendens* GV-12 followed by *A. ascendens* GV-8 yielded an acetic acid concentration of 408.09 mg/100 mL. And, the ethyl acetate concentration was approximately 5-fold higher than the *A. ascendens* GV-8 followed by *A. pasteurianus* GV-22 combination at pH 3.0 and 30 °C. This is presumed to be due to the more active volatilization and hydrolysis of aroma compounds at 30 °C than at 20 °C. However, because this study was limited to quality analyses under restricted fermentation conditions, pilot-scale validation is required. Future work should include sensory panel testing and analyses of physiological activity and metabolic pathways to elucidate mechanisms by which sequential starter fermentation enhances flavor and functionality. Overall, the results indicate that sequential AAB starter technology can be applied in the industry to produce vinegar with enriched aroma compounds.

## Figures and Tables

**Table 1:** Changes in organic acid content of black barley vinegar during sequential fermentation by stepwise inoculation of multiple AAB.

Sample	Organic Acid (mg/100 mL)					Total
	Citric Acid	Malic Acid	Succinic Acid	Lactic Acid	Acetic Acid	
BBV12-8 (day 0)	139.89 ± 0.49 ^b 1^	8.32 ± 0.12 ^b^	25.57 ± 0.87 ^a^	12.04 ± 0.51 ^a^	39.79 ± 3.09 ^b^	225.60 ± 5.08
BBV12-8 (day 10)	92.48 ± 0.24 ^d^	6.93 ± 0.04 ^c^	21.23 ± 0.06 ^b^	1.84 ± 0.01 ^b^	408.09 ± 1.45 ^a^	530.56 ± 1.81
BBV8-22 (day 0)	140.71 ± 0.35 ^a^	8.45 ± 0.10 ^b^	26.00 ± 0.04 ^a^	12.12 ± 0.05 ^a^	39.26 ± 0.05 ^b^	226.54 ± 0.59
BBV8-22 (day 10)	93.34 ± 0.06 ^c^	10.45 ± 0.15 ^a^	21.52 ± 0.83 ^b^	1.52 ± 0.25 ^b^	423.53 ± 25.74 ^a^	550.36 ± 27.02

^1^ The values are means ± SD (*n* = 3); different letters within the same column indicate a significant difference (*p* < 0.05) by Duncan’s multiple range test.

**Table 2:** Identification of major metabolites contributing to differentiation among black barley vinegar groups.

RT (min)	Compound	VIP ^1^	*p*-Value ^2^
2.72	Acetic acid, ethyl ester	1.44	5.24 × 10^−6^
3.34	Ethanol	0.95	1.45 × 10^−6^
3.71	Butanoic acid, ethyl ester	0.84	3.39 × 10^−3^
3.85	Acetic acid, propyl ester	1.29	3.50 × 10^−7^
4.52	Isobutyl acetate	1.24	9.04 × 10^−9^
6.37	2-Methyl-1-propanol	0.99	5.50 × 10^−5^
6.91	3-Methylbutyl acetate	1.28	2.21 × 10^−8^
7.42	cis-Ocimene	0.82	2.39 × 10^−1^
8.78	dl-Limonene	0.76	4.00 × 10^−1^
9.32	2-Methyl-1-butanol	1.09	6.46 × 10^−7^
9.91	Hexanoic acid, ethyl ester	0.92	7.69 × 10^−6^
11.23	3-Methoxy-1,2-propanediol	0.93	3.30 × 10^−7^
11.23	3-Hydroxy-2-butanone	0.93	3.38 × 10^−7^
12.43	5-Ethyl-2-nonanol	0.93	1.31 × 10^−1^
12.93	Ethyl 2-hydroxypropanoate	0.94	2.71 × 10^−7^
15.39	Acetic acid	0.93	5.15 × 10^−7^
16.44	Benzaldehyde	1.02	8.76 × 10^−5^
17.02	Isobutyric acid	0.93	7.42 × 10^−6^
17.80	Caryophyllene	0.73	4.47 × 10^−1^
18.99	Methyl salicylate	0.71	3.02 × 10^−1^
19.25	Acetic acid, 2-phenylethyl ester	1.05	2.60 × 10^−7^
19.91	Benzeneethanol	0.93	1.43 × 10^−3^

^1^ Variable importance in the projection (VIP) values were determined using partial least squares discriminant analysis (PLS-DA). ^2^ *p*-values (*p* < 0.05) were analyzed using one-way analysis of variance (ANOVA).

**Table 3:** Changes in major volatile compounds of black barley vinegar during sequential fermentation by stepwise inoculation of multiple AAB.

Group	Compound	BBV12-8 (Day 0)	BBV12-8 (Day 10)	BBV8-22 (Day 0)	BBV8-22 (Day 10)
Esters	Acetic acid, ethyl ester	54.21 ± 7.08 ^b 2^	106.95 ± 9.00 ^a^	54.73 ± 4.74 ^b^	18.65 ± 2.14 ^c^
	Butanoic acid, ethyl ester	0.09 ± 0.04	N.D. ^1^	0.09 ± 0.01	N.D.
	Acetic acid, propyl ester	0.19 ± 0.01 ^b^	1.04 ± 0.10 ^a^	0.16 ± 0.03 ^b^	0.14 ± 0.01 ^b^
	Isobutyl acetate	0.38 ± 0.03 ^b^	4.87 ± 0.31 ^a^	0.46 ± 0.02 ^b^	0.76 ± 0.11 ^b^
	3-Methylbutyl acetate	2.32 ± 0.19 ^b^	20.64 ± 1.53 ^a^	2.30 ± 0.04 ^b^	2.36 ± 0.30 ^b^
	Hexanoic acid, ethyl ester	0.06 ± 0.01	N.D.	0.08 ± 0.01	N.D.
	Ethyl 2-hydroxypropanoate	0.17 ± 0.02	N.D.	0.19 ± 0.01	N.D.
	Acetic acid, 2-phenylethyl ester	N.D.	0.20 ± 0.02 ** ^3^	N.D.	0.08 ± 0.02
Alcohols	Ethanol	31.15 ± 3.74 ^a^	5.74 ± 0.54 ^b^	27.82 ± 2.46 ^a^	0.56 ± 0.14 ^b^
	2-Methyl-1-propanol	1.22 ± 0.13 ^a^	0.71 ± 0.09 ^b^	1.08 ± 0.07 ^a^	0.42 ± 0.01 ^c^
	2-Methyl-1-butanol	13.50 ± 0.93 ^a^	8.25 ± 0.46 ^b^	12.89 ± 0.53 ^a^	3.36 ± 0.44 ^c^
	5-Ethyl-2-nonanol	0.11 ± 0.02 ^ab^	0.17 ± 0.05 ^a^	0.11 ± 0.01 ^ab^	0.10 ± 0.00 ^b^
	Benzeneethanol	0.26 ± 0.02 ^b^	0.22 ± 0.01 ^bc^	0.31 ± 0.02 ^a^	0.18 ± 0.03 ^c^
Acids	Acetic acid	2.13 ± 0.34 ^b^	26.14 ± 2.52 ^a^	2.07 ± 0.25 ^b^	24.51 ± 2.31 ^a^
	Isobutyric acid	0.06 ± 0.00 ^b^	0.15 ± 0.02 ^a^	N.D.	0.16 ± 0.01 ^a^
Terpenes	cis-Ocimene	0.18 ± 0.07 ^a^	0.16 ± 0.07 ^a^	0.18 ± 0.03 ^a^	0.08 ± 0.02 ^a^
	dl-Limonene	0.16 ± 0.08 ^a^	0.22 ± 0.08 ^a^	0.20 ± 0.03 ^a^	0.11 ± 0.06 ^a^
	Caryophyllene	0.69 ± 0.10 ^a^	0.85 ± 0.19 ^a^	0.69 ± 0.15 ^a^	0.64 ± 0.02 ^a^
Others	3-Methoxy-1,2-propanediol	0.29 ± 0.03	N.D.	0.29 ± 0.03	N.D.
	3-Hydroxy-2-butanone	N.D.	1.61 ± 0.17	N.D.	1.55 ± 0.14
	Benzaldehyde	0.05 ± 0.01 ^b^	0.08 ± 0.02 ^b^	N.D.	0.13 ± 0.01 ^a^
	Methyl salicylate	1.05 ± 0.57 ^a^	1.41 ± 0.50 ^a^	0.70 ± 0.06 ^a^	0.77 ± 0.09 ^a^

^1^ N.D., not detected. ^2^ The values are means ± SD (*n* = 3); different letters within the same row indicate a significant difference (*p* < 0.05) by Duncan’s multiple range test. ^3^ A double asterisk (**) within the same row indicates a significant difference at *p* < 0.01 by Student’s *t*-test.

## Data Availability

The original contributions presented in this study are included in the article and Supplementary Materials. Further inquiries can be directed to the corresponding author.

## Supplementary Materials

The following supporting information can be downloaded at: https://www.mdpi.com/article/10.3390/microorganisms13112576/s1, Table S1: Free amino acid composition of black barley vinegar during sequential fermentation.
