# Supplementary material for: Development of Sequential Fermentation Starters and Comparison of Quality Characteristics for Black Barley Vinegar Production
Source: Microorganisms. 2025 Nov 12;13(11):2576. doi: 10.3390/microorganisms13112576 (PMC12654374; doi:10.3390/microorganisms13112576)
Supplement: Supplementary file 1 [file microorganisms-13-02576-s001.zip › microorganisms-3936801-supplementary.pdf]

Development of Sequential Fermentation Starters and Comparison of Quality Characteristics for Black Barley Vinegar Production

Soo-Young Lee, Hyun-wook Jang and Hee-Min Gwon *

Fermented and Processed Food Research Division, Department of Food Sciences, NICS, RDA, Wanju 55365, Republic of Korea

* Correspondence: vitamin89@korea.kr; Tel.: +82-63-238-3613; Fax: +82-63-238-3843

Supplementary files:

Table S1. Free amino acid composition of black barley vinegar during sequential fermentation.

Table S1. Free amino acid composition of black barley vinegar during sequential fermentation.

Free amino acid (ug/mL) [1]	BBV12-8 (day 0)	BBV12-8 (day 10)	BBV8-22 (day 0)	BBV8-22 (day 10)
a-ABA	116.51 ± 5.51	N.D. [2]	75.22 ± 48.12	N.D.
Ala	651.70 ± 16.30 [ab] [3]	389.91 ± 27.60 [bc]	797.36 ± 215.95 [a]	328.30 ± 19.21 [c]
Arg	529.68 ± 10.16 [a]	277.15 ± 17.84 [b]	607.56 ± 115.81 [a]	288.61 ± 6.24 [b]
b-AiBA	90.88 ± 1.72 [a]	42.92 ± 3.23 [b]	51.90 ± 30.93 [b]	45.03 ± 3.10 [b]
Cys	125.63 ± 5.19 [a]	N.D.	93.66 ± 37.40 [a]	38.91 ± 4.95 [b]
Cysthi	36.91 ± 1.20 [a]	17.78 ± 2.53 [b]	34.08 ± 1.89 [a]	15.98 ± 3.73 [b]
EOHNH2	26.10 ± 1.13 [a]	7.52 ± 2.68 [b]	14.78 ± 8.94 [b]	4.91 ± 2.33 [b]
g-ABA	155.56 ± 3.41 [a]	40.83 ± 4.96 [b]	104.82 ± 63.57 [ab]	37.75 ± 5.14 [b]
Glu	537.74 ± 2.15 [a]	189.07 ± 7.99 [b]	661.90 ± 171.97 [a]	148.48 ± 5.96 [b]
Gly	159.75 ± 107.60 [a]	37.99 ± 42.31 [a]	11.77 ± 6.01 [a]	60.61 ± 41.67 [a]
His	185.10 ± 5.97 [a]	92.60 ± 7.94 [b]	138.33 ± 56.19 [ab]	109.25 ± 6.50 [b]
Hylys	8.37 ± 0.13 [a]	6.16 ± 0.35 [a]	5.55 ± 1.97 [a]	N.D.
Hypro	294.02 ± 2.32 [ab]	N.D.	430.56 ± 189.11 [a]	9.65 ± 0.44 [b]
Ile	272.41 ± 7.26 [a]	92.30 ± 7.86 [b]	215.61 ± 70.90 [a]	94.57 ± 8.67 [b]
Leu	655.99 ± 11.69 [a]	209.59 ± 12.75 [b]	522.25 ± 180.27 [a]	203.30 ±8.01 [b]
Lys	437.61 ± 9.82 [a]	221.91 ± 14.13 [b]	407.13 ± 32.20 [a]	238.57 ±10.08 [b]
Met	200.92 ± 5.48 [a]	81.41 ± 6.68 [b]	176.02 ± 28.41 [a]	75.63 ± 7.29 [b]
NH3	18.36 ± 1.71 [a]	4.50 ± 2.49 [b]	19.37 ± 2.01 [a]	3.35 ± 2.93 [b]
Orn	36.13 ± 0.38 [b]	61.92 ± 4.70 [a]	23.83 ± 13.50 [b]	59.19 ± 4.36 [a]
Phe	456.32 ± 5.30 [a]	155.45 ± 9.12 [b]	361.94 ± 126.82 [a]	154.60 ± 7.08 [b]
Pro	445.98 ± 13.26 [a]	159.39 ± 11.79 [b]	304.48 ± 186.88 [ab]	159.56 ± 6.48 [b]
P-Ser	28.96 ± 1.33 [a]	25.51 ± 1.52 [bc]	28.09 ± 0.12 [ab]	23.80 ± 0.60 [c]
Sar	74.88 ± 1.76 [a]	59.23 ± 7.65 [a]	69.43 ± 2.35 [a]	48.00 ± 17.66 [a]

Tau	38.93 ± 1.97 [a]	46.37 ± 4.10 [a]	26.24 ± 16.13 [a]	45.36 ± 2.09 [a]
Tyr	376.20 ± 14.90 [a]	134.31 ± 12.88 [b]	252.78 ± 155.07 [ab]	135.57 ± 12.10 [b]
Urea	83.90 ± 15.48 [a]	119.53 ± 42.90 [a]	95.13 ± 0.41 [a]	68.03 ± 7.65 [a]
Val	309.73 ± 8.48 [a]	128.63 ± 8.53 [b]	282.06 ± 38.08 [a]	113.22 ± 5.22 [b]

[1] Symbols: α-ABA, L-α-Amino-n-butyric acid; Ala, L-Alanine; Arg, L-Arginine; β-AiBA, β-Aminoisobutyric acid; Cys, L-Cystine; Cysthi, L-Cystathionine; $EOHNH_2$, Ethanolamine; γ-ABA, γ-Aminobutyric acid; Glu, L-Glutamic acid; Gly, L-Glycine; His, L-Histidine; Hylys, D,L- & allo-Hydroxylysine; Hypro, Hydroxyproline; Ile, L-Isoleucine; Leu, L-Leucine; Lys, L-Lysine; Met, L-Methionine; NH_3, Ammonia; Orn, L-Ornithine; Phe, L-Phenylalanine; Pro, L-Proline; P-Ser, D,L-O-Phosphoserine; Sar, Sarcosine; Tau, Taurine; Tyr, L-Tyrosine; Urea, Urea; Val, L-Valine.

[2] N.D., not detected. [3] The values are means ± SD (n = 3); different letters within the same column indicate a significant difference ($p < 0.05$) by Duncan's multiple range test.
